# Targeting Claudin18.2 for cancer theranostics: From molecular imaging to precision therapy

**DOI:** 10.1016/j.isci.2025.113491

**Published:** 2025-09-02

**Authors:** Yongshun Liu, Wenpeng Huang, Jessica C. Hsu, Zhaonan Sun, Weibo Cai, Lei Kang

**Affiliations:** 1 Department of Medical Imaging, Peking University First Hospital, Beijing 100034, China; 2 Department of Nuclear Medicine, Peking University First Hospital, Beijing 100034, China; 3 Departments of Radiology and Medical Physics, University of Wisconsin - Madison, Madison, WI 53705, USA

**Keywords:** medical imaging, oncology, therapeutics

## Abstract

Claudin18.2 (CLDN18.2), a specific tight junction protein isoform, is minimally expressed in normal gastric mucosa but aberrantly overexpressed in various cancers. It plays a key role in regulating tumor cell differentiation, proliferation, and migration, making it an attractive therapeutic target, especially in gastric cancer. Moreover, molecular imaging techniques such as immuno-positron emission tomography, immuno-single photon emission computed tomography, and near-infrared fluorescence imaging enable non-invasive evaluation of CLDN18.2 expression, improving diagnosis and guiding personalized treatment. This review summarizes recent advances in CLDN18.2-targeted therapies and molecular imaging for cancer management. We outline the biomarker’s biological functions and signaling pathways across cancers, highlighting the development of precision therapeutics. We also discuss applications and limitations of CLDN18.2-targeted theranostics in digestive malignancies and address clinical translation challenges and future directions.

## Graphical abstract

**Figure:**
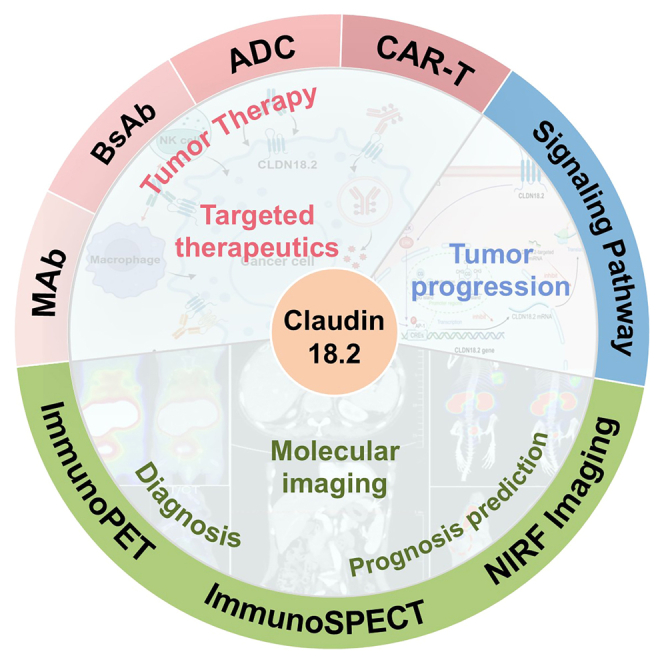

## Introduction

Claudins (CLDNs) are a family of transmembrane proteins localized at the tight junctions of epithelial and endothelial cells, first discovered in 1998.^1^ CLDN proteins, with a molecular weight of 20–27 kDa, are characterized by four transmembrane domains and two extracellular loops, with both NH2- and COOH-termini located intracellularly.^2^ CLDNs are vital in maintaining cell-cell adhesion, regulating epithelial polarity, and controlling paracellular permeability across tissue barriers.^3^^,^^4^ CLDN18, classed as a non-classical claudin, is a key component of tight junctions in lung and gastric epithelial cells.^5^ The human *CLDN18* gene is genomically positioned at chromosome 3q22 and spans approximately 35 kilobases, comprising six coding exons interspersed with five non-coding introns.^6^ Through alternative splicing, it produces two distinct protein isoforms: CLDN18.1, predominantly expressed in lung tissue, and CLDN18.2, restricted to differentiated epithelial cells of the gastric mucosa.^7^

Under normal physiological states, CLDN18.2 is expressed at low levels, where it functions to protect the gastric mucosa and inhibit acid secretion.^2^ However, its aberrant overexpression has been reported in a broad range of malignancies, including pancreatic, colorectal, breast, and hepatocellular carcinomas.^8^^,^^9^ In these contexts, CLDN18.2 contributes to tumor progression by regulating cell differentiation, proliferation, and migration.^10^ Thus, upregulated expression in tumors positions CLDN18.2 as an attractive therapeutic target, particularly in gastric cancer. The US Food and Drug Administration (FDA) approval of zolbetuximab, the first CLDN18.2-targeted monoclonal antibody, has marked a milestone in first-line gastric cancer treatment, highlighting the clinical relevance of CLDN18.2. Moreover, growing evidence indicates that CLDN18.2 expression correlates strongly with both patient prognosis and therapeutic response across multiple malignancies.^11^^,^^12^^,^^13^ Accurate quantification of CLDN18.2 expression is thus essential for stratifying patients who may benefit from targeted therapies. While immunohistochemistry (IHC) remains the standard method for assessing CLDN18.2 in clinical settings, it has notable limitations, including invasiveness, sampling variability, and inability to capture heterogeneous expression patterns across tumor sites.^14^

Molecular imaging offers a powerful non-invasive alternative for visualizing and quantifying CLDN18.2 expression *in vivo*, enabling real-time assessment of target distribution and changes over the course of treatment.^15^ Radiopharmaceutical-based imaging, particularly immuno-positron emission tomography (immunoPET) and immuno-single photon emission computed tomography (immunoSPECT), has emerged as a leading strategy. These modalities combine the high sensitivity of nuclear imaging with the specificity of antibody-based targeting and have demonstrated substantial clinical utility in gastrointestinal cancers.^16^ Additionally, optical imaging methods, such as near-infrared fluorescence (NIRF) imaging, are increasingly used to facilitate intraoperative guidance and improve surgical outcomes.^17^

This review presents a comprehensive synthesis of current advances in CLDN18.2-targeted therapeutics and molecular imaging technologies for cancer management. We first briefly outline the functional significance and underlying oncogenic pathways associated with CLDN18.2 across multiple cancer types. We then highlight emerging therapeutic strategies targeting this biomarker, followed by an in-depth discussion of novel applications and limitations of CLDN18.2-targeted theranostics in various cancers represented by digestive system tumors. Finally, we address the challenges associated with clinical translation and propose future research directions to advance CLDN18.2-targeted precision medicine further. Our analysis establishes novel frameworks for CLDN18.2-targeted molecular imaging and addresses underexplored dimensions in current biomarker-guided imaging literature.

## Significance of Claudin18.2 in solid tumors

CLDN18.2, a differentiation-associated transmembrane protein, exhibits abnormal expression across a wide range of malignancies. Its function in oncogenesis is context-dependent, engaging in either tumor-promoting signaling cascades or growth-inhibitory pathways, depending on the tissue-specific molecular environment.^8^^,^^9^ While this protein generally promotes cell proliferation, differentiation, and migration, it displays tumor-suppressive activity in certain subtypes of gastric cancer and lung adenocarcinoma.^18^^,^^19^^,^^20^ In this section, we outline the expression patterns and clinical relevance of CLDN18.2 in key tumor types, including gastric cancer, pancreatic cancer, cholangiocarcinoma (CCA), and lung cancer.

### Gastric cancer

Gastric cancer remains a major public health concern, ranking among the highest in both incidence and mortality among digestive tract malignancies.^21^ CLDN18.2 expression varies widely among molecular subtypes of gastric cancer, with higher levels commonly found in diffuse-type adenocarcinomas and HER2-amplified tumors.^18^^,^^19^ Conversely, CLDN18.2 expression is notably downregulated in certain subtypes, such as gastric stromal tumors. Wang et al.^12^ reported lower CLDN18.2 expression levels in Borrmann type 3 or 4 and moderately to well-differentiated gastric cancers. Overall, CLDN18.2 expression positively correlates with tumor differentiation and negatively correlates with perineural infiltration.^22^^,^^23^ Several studies have linked reduced CLDN18.2 expression to poorer overall survival (OS),^11^^,^^23^^,^^24^ while others suggest that positive CLDN18.2 expression may predict worse clinical outcomes.^12^ However, multiple reports have found no significant association between CLDN18.2 levels and OS.^25^^,^^26^^,^^27^ These discrepancies are likely attributable to methodological differences, such as variations in patient selection criteria, which may lead to substantial biological heterogeneity and limit the generalizability of findings.

### Pancreatic cancer

Pancreatic cancer is a highly aggressive malignancy with a poor prognosis, largely due to late-stage diagnosis and limited effective treatment options.^28^ While CLDN18.2 is minimally expressed in normal pancreatic tissue, it is markedly overexpressed in pancreatic tumors.^29^ Studies have reported CLDN18.2 expression in 59.2% of primary pancreatic adenocarcinomas, 65.7% of hepatic metastases, and 69.4% of lymph node metastases.^29^^,^^30^ Beyond its expression prevalence, CLDN18.2 holds clinical and pathological significance. In gastric cancer, its expression inversely correlates with neural invasion and is significantly associated with tumor differentiation status.^22^^,^^23^ For instance, CLDN18.2 was detected in 59.0% of G3-grade tumors versus 37.7% of G1/2-grade tumors.^22^ In pancreatic cancer, higher CLDN18.2 expression is preferentially associated with well-differentiated carcinomas and improved patient survival compared to low-expressing cases.^31^ Collectively, these findings underscore CLDN18.2 as a multifaceted biomarker with both diagnostic and prognostic potential.

### CCA

CCA, the second most common hepatic malignancy globally, presents significant clinical challenges due to its aggressive nature and high mortality rate.^32^ It is categorized into three primary subtypes: intrahepatic, perihilar, and distal CCA. CLDN18 is overexpressed in both intrahepatic and extrahepatic CCA subtypes, with upregulation detectable as early as the biliary intraepithelial neoplasia stage.^33^ Functionally, CLDN18 enhances the proliferative and invasive potential of CCA cells, supporting its role as a tumor-promoting factor and potential pathogenic driver.^34^ Beyond its role in tumor initiation, elevated CLDN18 expression is linked with advanced disease stage, including lymph node metastasis (LNM) and poorer OS in intrahepatic CCA.^33^ This dual involvement in tumorigenesis and metastasis underscores CLDN18’s potential as a theranostic target for precision medicine in biliary tract malignancies.

### Lung cancer

Lung cancer remains one of the most prevalent malignancies worldwide, with significantly lower OS rates relative to other major cancers.^35^ Transcriptomic analyses have shown a marked reduction in CLDN18 expression in lung adenocarcinoma tissues relative to normal pulmonary tissue.^20^ Survival data further indicate that high CLDN18.2 expression is associated with favorable clinical outcomes, highlighting its potential as a prognostic biomarker.^20^ An independent study reported significantly higher CLDN18.2 expression in mucinous adenocarcinoma compared to non-mucinous subtypes.^36^ An elevated level of CLDN18.2 is correlated with reduced tumor size, decreased pleural invasion rates, and earlier nodal metastasis stages, suggesting its potential as a predictive marker for tumor aggressiveness in lung adenocarcinoma.^36^

## Molecular mechanism of Claudin18.2 in cancers

**Figure 1:**
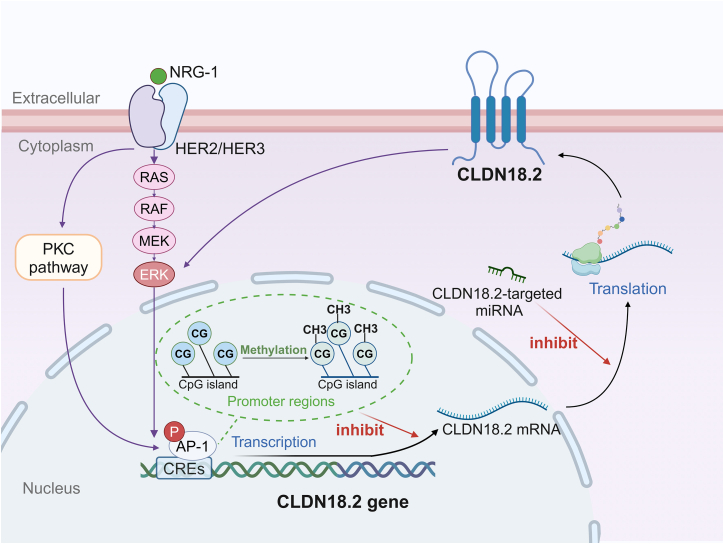
Signaling pathways that govern Claudin18.2 expression NRG-1*,* neuregulin 1. Created in https://BioRender.com.

Understanding the molecular mechanisms underlying tumor-associated targets is critical for advancing targeted therapies and imaging techniques. Current research on CLDN18.2 mainly explores its upstream signaling pathways (Figure 1), providing critical insights into the origins and regulatory mechanisms of its dysregulation.

Gene transcription regulates the expression of CLDN18.2. CpG islands, genomic regions enriched in cytosine-phosphate-guanine dinucleotide motifs, are commonly located in gene promoters and exonic sequences.^37^ Methylation of promoter-associated CpG sites in the *CLDN18.2* gene inhibits the binding of transcription factor *cis*-regulatory elements (CREs), thereby silencing gene expression via epigenetic mechanisms.^38^

Several signaling pathways also modulate CLDN18.2 transcription, including the protein kinase C (PKC), mitogen-activated protein kinase (MAPK), and human epidermal growth factor receptor (HER) pathways. PKC is a critical intracellular signaling cascade that orchestrates transcriptional activity, secretory, cellular proliferation, and immune responses.^39^ Activation of the PKC signaling pathway markedly upregulates CLDN18.2 expression, whereas its inhibition suppresses this effect, highlighting PKC as a positive regulator of CLDN18.2 transcription.^38^ Mechanistically, PKC activation induces phosphorylation of activator protein 1 (AP-1), which then binds to the cAMP response element in the CLDN18.2 promoter, enhancing mRNA synthesis and protein expression.^40^

The MAPK family, composed of conserved serine/threonine kinases, mediates extracellular signal transduction to the nucleus.^41^ It includes four main subgroups: extracellular signal-regulated kinase (ERK), ERK5, c-Jun N-terminal kinase, and p38. The ERK/MAPK pathway promotes CLDN18.2 transcription via phosphorylation-driven activation of AP-1, facilitating its binding to regulatory promoter elements.^40^ Furthermore, the SPAK-p38 MAPK pathway has been implicated in suppressing CLDN18 expression and disrupting alveolar epithelial barrier integrity under hypoxic conditions.^42^

The HER family comprises four transmembrane receptor tyrosine kinases: epidermal growth factor receptor (EGFR, also known as HER1), HER2, HER3, and HER4.^43^ HER receptor regulates cell differentiation, proliferation, and motility through the protein kinase B (PKB/AKT) and MAPK cascades. EGFR/ERK signaling has been shown to drive CLDN18 transcription, while increased CLDN18 expression can, in turn, activate ERK1/2, forming a feedforward loop that enhances tumor cell proliferation and invasion, particularly in CCA.^34^ HER2/HER3 signaling also modulates CLDN18 expression and barrier function, although its molecular mechanism is not fully understood. Furthermore, this signaling axis can suppress CLDN18 protein synthesis and disrupt membrane trafficking, contributing to alveolar-capillary barrier dysfunction in acute respiratory distress syndrome (ARDS).^44^

The CLDN18 transcript is subject to post-transcriptional regulation by microRNAs (miRNAs), a class of non-coding RNA that orchestrate gene expression by mediating mRNA stability and translation through sequence-specific binding.^45^ The miRNAs bind to complementary regions of target mRNAs, leading to transcript degradation or translational repression, thereby fine-tuning gene expression at the post-transcriptional level.^46^ Studies have demonstrated that miR-1303 and miR-767-3p substantially downregulate CLDN18 expression by directly targeting its mRNA, resulting in reduced cell proliferation, motility, and metastatic potential.^47^^,^^48^

Despite its recognized importance, the precise mechanisms through which CLDN18.2 influences tumor progression remain elusive. Current evidence suggests that CLDN18.2 may modulate several signaling pathways, including the programmed cell death protein-1 (PD-1), Wnt, and ERK pathways, thereby contributing to tumor development and progression.^34^^,^^49^^,^^50^ Conversely, tumor-suppressive functions of CLDN18 have been observed in certain subtypes of gastric and lung cancers.^10^ However, robust evidence supporting the direct correlation between CLDN18 downregulation and gastric carcinogenesis remains limited. These effects may stem from its ability to modulate Wnt and Notch signaling pathways and mediate feedback loops involving the transcriptional co-activator Yes-associated protein (YAP).^10^^,^^51^^,^^52^ Given the limited mechanistic understanding, further research is needed to delineate the dual role of CLDN18.2 in cancer and to avoid the unintended pro-tumor effects of CLDN18.2-targeted drugs in malignant tumors where CLDN18.2 acts as a tumor suppressor.

## Claudin18.2 as a cancer therapeutic target

**Figure 2:**
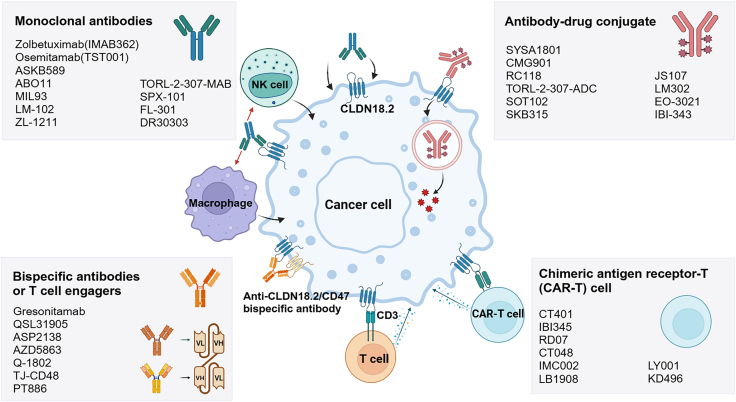
The CLDN18.2-targeted therapeutic agents NK, natural killer. Created in https://BioRender.com.

CLDN18.2 has emerged as a promising therapeutic target in digestive system cancers, drawing growing interest in the field of precision oncology. Current investigational CLDN18.2-targeted agents include monoclonal antibodies (mAbs), bispecific antibodies (BsAbs), antibody-drug conjugates (ADCs), and chimeric antigen receptor T (CAR-T) cell therapies (Figure 2). This section provides an overview of the key concepts and recent advancements in these therapeutic strategies.

### mAb

mAbs are immunoglobulins derived from a single B cell clone, exerting their biological activity through specific recognition of a single antigenic epitope.^53^ Owing to their high specificity and binding affinity, mAbs are integral to the molecularly targeted therapy of malignancies and immune-related diseases. Several mAbs targeting CLDN18.2 are currently in clinical development, including IMAB362, TST001, ASBB589, AB011, MIL93, and M108.

Zolbetuximab (IMAB362) is the first chimeric immunoglobulin G1 (IgG1) mAb developed to specifically target CLDN18.2.^54^ It selectively binds to CLDN18.2 expressed on the surface of tumor cells and activates multiple antitumor mechanisms, including antibody-dependent cellular cytotoxicity (ADCC), complement-dependent cytotoxicity (CDC), apoptosis induction, and proliferation suppression.^55^ Preclinical studies have demonstrated its potent antitumor activity. Clinical evaluation in phase I/II trials has confirmed its efficacy and safety in patients with gastric and gastroesophageal junction (GEJ) cancers.^56^ In the phase III SPOTLIGHT trial (NCT03504397), which enrolled 565 patients with CLDN18.2-positive, HER2-negative gastric or GEJ adenocarcinoma, zolbetuximab combined with mFOLFOX6 significantly improved progression-free survival (PFS: 10.6 vs. 8.7 months) and OS (18.2 vs. 15.5 months) compared to chemotherapy alone. Similarly, the phase III GLOW trial (NCT03653507) demonstrated that zolbetuximab plus CAPOX significantly prolonged PFS (7.2 vs. 5.3 months) and OS (14.6 vs. 12.2 months) in first-line treatment of unresectable/metastatic gastric or GEJ cancer. Based on these pivotal findings, the US FDA approved zolbetuximab in combination with fluoropyrimidine and platinum-based chemotherapy as a first-line treatment for adult patients with HER2-negative, CLDN18.2-positive, locally advanced or metastatic gastric and GEJ adenocarcinoma.^57^ This approval marks the clinical integration of CLDN18.2-targeted therapy. Ongoing phase III clinical trials are further evaluating its efficacy in patients with moderate-to-high CLDN18.2 expression, defined as >70% tumor cell positivity.

Osemitamab (TST001) is the second CLDN18.2-targeted therapeutic candidate to enter clinical development. This humanized IgG1 mAb exhibits high-affinity binding to CLDN18.2 and demonstrates stronger ADCC and CDC activity than zolbetuximab in preclinical studies.^58^ Furthermore, osemitamab demonstrated greater anti-tumor efficacy than zolbetuximab in patient-derived xenograft (PDX) models. When combined with chemotherapy, TST001 showed synergistic antitumor effects across various tumor models.^8^ Phase I clinical studies (NCT04396821 and NCT04495296) confirmed the safety of TST001 as monotherapy and in combination with chemotherapy. Data from the 2024 phase I/IIa G cohort study further supported the safety and feasibility of osemitamab in combination with nivolumab and CAPOX for advanced gastric cancer and GEJ adenocarcinoma, providing a strong rationale for further clinical development.

ASKB589 is a second-generation humanized CLDN18.2-targeted mAb, designed to enhance immune-mediated tumor cell killing.^59^ It promotes T cell infiltration and antigen presentation, thereby improving the effectiveness of immune checkpoint inhibitors. In the Ib/II trial (NCT05632939), ASKB589 combined with chemoimmunotherapy achieved an 80% objective response rate (ORR) and 100% disease control rate (DCR) in 45 patients with high CLDN18.2 expression. This regimen is now undergoing global multicenter phase III clinical validation as a frontline therapy for CLDN18.2-positive advanced gastric cancer.

Together, mAb-based therapies represent a promising new class of precision medicine targeting CLDN18.2. Notably, long-term CLDN18.2-targeted therapy such as zolbetuximab may lead to gastric mucosal barrier dysfunction and induce chronic immune-mediated damage.^60^ In addition, changes in antigen expression or binding, impaired antibody-dependent cytotoxicity, and anti-apoptotic effects may all lead to resistance to CLDN18.2-targeted mAbs.^61^ Strategies such as using intelligent responsive materials for precise drug delivery and synthesizing lethal combination therapies can be considered to overcome drug resistance.

### BsAb

BsAbs are artificially engineered molecules designed to simultaneously bind two distinct antigens or epitopes.^62^ This dual-targeting capability has shown clinical efficacy in both cancer and autoimmune diseases. Preclinical studies have demonstrated that BsAbs targeting CLDN18.2 exhibit potent tumor-suppressive effects. For example, a CLDN18.2-CD3 BsAb suppressed tumor growth *in vitro* and in gastric and pancreatic cancer PDX models.^63^ Similarly, the CLDN18.2-CD28 BsAb enhances the infiltration and activation of CD8^+^ T cells, reduces immunosuppressive cell populations, promotes the secretion of IFN-γ and TNF-α, and improves the overall tumor microenvironment.^64^

The dual-targeting ability of BsAbs can also reduce off-tumor toxicity in normal tissues with low expression of CLDN18.2 through specific enhancement mechanisms and dynamic pharmacokinetic regulation.^65^ The CLDN18.2-PDL1 BsAb, Q-1802, activates a dual immune response by blocking PD-1 signaling and mediating ADCC, thereby exhibiting high anti-tumor activity in preclinical models.^66^ Q-1802 has been evaluated in a phase I clinical trial (NCT04856150), where dose-escalation reached 20 mg/kg without dose-limiting toxicities. The most common adverse events were nausea, vomiting, and abdominal pain. The trial data indicate that Q-1802 exhibits a favorable safety profile, manageable toxicities, and preliminary evidence of antitumor efficacy. However, the current evidence base remains insufficient to extrapolate these pharmacodynamic outcomes to broader, more diverse patient cohorts. Another bispecific agent, Gresonitamab (AMG-910), is a bispecific T cell engager (BiTE) that directs CD3^+^ T cells to CLDN18.2^+^ tumor cells and is under phase I trial (NCT04260191) for advanced gastric cancer and GEJ adenocarcinoma. Additional BsAbs targeting CLDN18.2 and CD3, including AZD5863 (NCT06005493) and ASP2138 (NCT05365581), are also in early-phase clinical trials.

Despite their therapeutic promise, BsAbs are more complex to develop than mAbs, with unique technical challenges in design, manufacturing, and clinical translation. Safety concerns, particularly cytokine release syndrome (CRS), remain a critical consideration in ongoing clinical evaluations.^67^ In addition, BsAbs may contribute to the progression of immune-related adverse events (irAEs) during long-term treatment through the cumulative effects of sustained immune activation, additive toxicity from target cross-reactivity, and prolonged competition in metabolic pathways.^68^^,^^69^

### ADC

ADCs exhibit significant therapeutic potential in cancer treatment by coupling mAbs specific to CLDN18.2 with cytotoxic payloads, enabling selective drug delivery to tumors with high CLDN18.2 expression.^70^ Several CLDN18.2-targeted ADC candidates have recently progressed through preclinical development and early-phase clinical trials, reflecting their growth relevance in precision oncology. Anti-CLDN18.2 antibody conjugated with a cleavable auristatin demonstrated robust cytotoxic effects in gastric and pancreatic cancer cell lines, as well as in PDX models.^63^ The agent exhibited a favorable safety profile, with no significant toxicity observed in gastric tissues.

SYSA1801 (EO3021) is an ADC comprising a CLDN18.2-targeting mAb linked to monomethyl auristatin E (MMAE) via a cleavable valine-citrulline linker.^71^ In an ongoing phase I clinical trial (NCT05009966), 33 patients with advanced, heavily pretreated CLDN18.2-positive gastric or pancreatic cancer received SYSA1801 monotherapy. Among 21 evaluable patients, the ORR was 38.1% with a DCR of 57.1%. In patients with advanced gastric cancer specifically, ORR and DCR rose to 47.1% and 64.7%, respectively. Common adverse events included nausea, vomiting, and dry eye syndrome, indicating minimal safety concerns.

CMG901 (AZD0901), the first ADC developed to target CLDN18.2, consists of a CLDN18.2-specific mAb, a cleavable linker, and an MMAE payload.^56^ Preclinical studies demonstrated that CMG901 exhibits potent cytotoxic effects against gastric cancer cells, with significantly enhanced antitumor efficacy compared to zolbetuximab analogs or its unconjugated counterpart. CMG901 maintained acceptable tolerability with a favorable safety margin in preclinical toxicology assessments.^72^ In a phase I clinical trial (NCT04805307), CMG901 demonstrated a 42% ORR in 113 heavily pretreated patients with advanced gastric or GEJ cancers. The most frequent treatment-emergent adverse events were anemia and neutropenia. CMG901 is being evaluated in a phase III trial (NCT06346392) for advanced CLDN18.2-positive gastric cancer and GEJ adenocarcinoma.^72^

A critical challenge in ADC development stems from off-target toxicity driven by non-specific antigen binding, premature payload release in circulation, and the bystander effect. Current mitigation strategies focus on optimizing linker stability and site-specific conjugation to improve targeting precision, engineering payloads with reduced bystander effect, and developing predictive computational models.

### CAR-T cell

CAR-T cell therapy involves genetically engineering autologous T cells to express synthetic receptors that recognize tumor-specific antigens, thus enabling targeted cytotoxicity.^73^ While this strategy has transformed the treatment landscape for hematologic cancers, its application in solid tumors remains limited due to key biological challenges. These include suboptimal target selection, antigen heterogeneity, and adverse reactions such as CRS and immune effector cell-associated neurotoxicity syndrome.^74^ Current mitigation strategies for CAR-T-associated CRS encompass coordinated approaches in cellular engineering, pharmacological modulation, real-time biomarker-guided monitoring, and refinement of dosing regimens.^75^

The restricted expression of CLDN18.2 in normal tissues and its selective overexpression in various gastrointestinal malignancies make it an attractive and clinically actionable target for CAR-T cell therapy. Leveraging humanized CLDN18.2-specific single-chain variable fragments (scFvs) derived from hu8E5 and hu8E5-2I antibodies, researchers have successfully engineered second-generation CAR-T constructs (hu8E5-28Z and hu8E5-2I-28Z), incorporating a CD28 co-stimulatory domain. These CAR-T cells specifically recognize CLDN18.2 with minimal cross-reactivity to CLDN18.1, thereby mitigating off-target toxicity risks.^76^ *In vitro* studies demonstrated selective lysis of CLDN18.2-positive tumor cells and target-dependent cytokine release, with negligible cytotoxicity toward CLDN18.2-negative cells. In PDX models of gastric cancer, these CAR-T cells significantly suppressed tumor growth and induced complete remission in some cases. Moreover, they exhibited sustained persistence, robust tumor infiltration, and tissue specificity, with minimal toxicity to adjacent normal gastric mucosa or distal organs. These findings support the feasibility, safety, and therapeutic potential of CLDN18.2-targeted CAR-T cell therapy in solid tumors.

Currently, CLDN18.2-targeted CAR T cell therapies have advanced to clinical trials. CT041 represents a second-generation CAR-T cell therapeutic agent engineered to specifically recognize the CLDN18.2 epitope, a tumor-associated antigen. In a phase I clinical trial (NCT03159819) involving subjects with metastatic CLDN18.2-positive gastric and pancreatic malignancies, CT041 achieved a 33.3% ORR and a median PFS of 130 days. A subsequent phase I trial (NCT03874897) reported an improved ORR of 48.6% across multiple cancer types and 57.1% specifically in gastric cancer. The DCR reached 73.0% in a pan-cancer cohort and 75% in patients with gastric cancer. An additional phase I clinical trial (NCT04581473) is currently ongoing to evaluate the efficacy and safety of CT041 in patients with gastric, pancreatic, and GEJ adenocarcinomas. These findings highlight the significant therapeutic promise of CT041 for CLDN18.2-positive tumors. In parallel, several ongoing early-phase trials are evaluating other CLDN18.2-targeted CAR T cell therapies. These include second-generation CAR-T constructs such as LB1908 (NCT05539430), incorporating a 4-1BB co-stimulatory domain; third-generation CAR-T candidates such as LY011 (NCT04966143; NCT04977193); and fourth-generation “TRUCK” CAR-T constructs designed for cytokine or chemokine secretion, including CT048 (NCT05393986) and RD07 (NCT05284968).

Among the four targeted therapeutic modalities, CAR-T cell therapy achieves the highest spatial resolution through physical intercellular contact. ADCs achieve secondary specificity through tumor microenvironment-responsive payload deployment mechanisms. BsAbs exhibit higher specificity than mAbs due to their dual-targeting design, and mAbs rely on the abundance of a single target and lack downstream regulation.

## Claudin18.2-targeted molecular imaging

Molecular imaging enables non-invasive and precise detection to assess the expression and distribution of CLDN18.2 using radiolabeled or fluorescently labeled molecular probes, serving as a complementary diagnostic tool to identify patients suitable for CLDN18.2-targeted therapy.^15^ Currently, CLDN18.2-targeted imaging is primarily based on nuclear medicine techniques, particularly immunoPET and immunoSPECT, which employ radiolabeled antibodies (e.g., with ^89^Zr and ^68^Ga) to visualize tumors with high sensitivity and specificity. Additionally, NIRF imaging has been employed for intraoperative visualization, offering real-time guidance during surgical interventions.

To systematically evaluate the current landscape of CLDN18.2-targeted molecular imaging in malignancies, we conducted a comprehensive literature search of PubMed/MEDLINE and the Cochrane Library through March 24, 2025 (Table 1). The search used the following terms: (A) “gastrointestinal” OR “digestive” OR “gastric” OR “pancreatic” OR “pancreas” OR “colorectal” OR “colon and rectum”; (B) “cancer” OR “carcinoma” OR “tumor” OR “tumor” OR “neoplasm”; (C) “Claudin18.2” OR “Claudin-18.2” OR “CLDN18.2” OR “Claudin18 isoform 2”; (D) “imaging” OR “image” OR “PET” OR “PET/CT” OR “SPECT” OR “fluorescence.” The search was restricted to English-language articles without temporal restrictions. Authors of inaccessible studies were not contacted for manual retrieval. The selected studies must meet the following criteria: rigorous methodological design, validated research outcomes, and publication in peer-reviewed journals indexed in the Science Citation Index database.

**Table 1:** Theranostic applications of different molecular imaging targeting Claudin18.2

Imaging	Tracer	Tracer type	Cancer type	Tumor model	Findings	Reference
ImmunoPET	^89^Zr-DFO-TST001/IgG	mAb	gastric cancer	BGC823^CLDN18.2^/BGC823 xenograft tumor	^89^Zr-DFO-TST001 probe demonstrates specific high uptake in CLDN18.2-positive tumors with significant differentiation from background signals	Chen et al.^77^
ImmunoPET	^124^I-5C9/IgG	mAb	gastric cancer	BGC823^CLDN18.2^/BGC823 xenograft tumor	^124^I-5C9 immunoPET imaging can detect subcutaneous CLDN18.2-positive tumors in mice	Zhao et al.^78^
ImmunoPET	^124^I-18B10(10L)	mAb	gastric/pancreatic cancer	MKN45^CLDN18.2^/MKN45 xenograft tumor and patients with gastric/pancreatic cancer	^124^I-18B10 (10L) has high affinity in CLDN18.2-positive cells and can display tumor lesions overexpressing CLDN18.2 through PET imaging in humans	Wang et al.^79^
ImmunoPET	^89^Zr-zolbetuximab ^89^Zr-hu7v3-Fc	mAb sdAb	gastric cancer	SUN-620 (CLDN18.2+) xenograft tumor	^89^Zr-hu7v3-Fc exhibited a faster tumor uptake, better tumor penetration, and higher tumor-to-muscle ratio than ^89^Zr-zolbetuximab	Zhong et al.^80^
ImmunoPET	^89^Zr-*anti*-CLDN18.2 VHH/VHH-ABD/VHH-Fc	sdAb	gastric/prostate cancer	CO-SNU620 (CLDN18.2+) and PC3 (CLDN18.2-) xenograft tumor	^89^Zr-*anti*-CLDN18.2 VHH-ABD/VHH-Fc demonstrated high tumor accumulation in PET imaging *ex vivo* and *in vivo*	Hu et al.^81^
ImmunoPET	^68^Ga/^64^Cu-NOTA-hu19V3 ^18^F-hu19V3	sdAb	gastric/colon cancer	CHO/CT26-CLDN18.2 xenograft tumor No.144 (CLDN18.2+) and No.490 (CLDN18.2-) PDX	these tracers can effectively visualize the expression of CLDN18.2 and provide complementary information for annotating CLDN18.2	Wei et al.^82^
ImmunoPET	^68^Ga-NC-BCH	sdAb	gastric/colon cancer	AGS^CLDN18.2^/AGS xenograft tumor and patients with gastric/colon cancer	^68^Ga-NC-BCH exhibits significant uptake in CLDN18.2-positive gastrointestinal tumors. ^68^Ga-NC-BCH PET/CT showed a higher T/NT ratio in detecting lymph nodes and peritoneal metastases than ^18^F-FDG PET/CT	Qi et al.^83^
ImmunoPET	^68^Ga-PMD22	sdAb	gastric/colorectal cancer	BGC823^18.2^/BGC823 xenograft tumor and patients with gastric/colorectal cancer	^68^Ga-PMD22 has high specific binding ability in CLDN18.2-positive tumors, and PET/CT imaging has high diagnostic accuracy in patients. SUVmax is highly correlated with IHC staining results	Wang et al.^84^
ImmunoPET	^68^Ga-DOTA-T37	peptide	gastric cancer	BGC823^CLDN18.2^/BGC823 xenograft tumor	^68^Ga-DOTA-T37 probe can specifically target CLDN18.2-positive tissues and tumors *in vivo*	Wang et al.^85^
ImmunoSPECT	^177^Lu-TST001	mAb	gastric cancer	BGC823^CLDN18.2^/BGC823 and AGS^CLDN18.2^/AGS xenograft tumor	The ^177^Lu-TST001 probe demonstrates specific high tumor uptake in CLDN18.2-positive tumors.	Zeng et al.^86^
ImmunoSPECT	^123^I/^131^I-IMAB362	mAb	gastric cancer	MKN45 (CLDN18.2+) and MKN28 (CLDN18.2-) xenograft tumor	^123^I/^131^I-IMAB362 can function as a theranostic pair for CLDN18.2-targeted SPECT/CT and RIT	Wu et al.^87^
ImmunoSPECT	^125^I-IMAB362	mAb	gastric cancer	MKN45^CLDN18.2^/MKN45 xenograft tumor	^125^I-IMAB362 has a high specific binding ability to MKN45-CLDN18.2 cells and shows long-term retention at the tumor	Wang et al.^88^
ImmunoSPECT	^99m^TC-PHG102	sdAb	gastric cancer AEG	BGC823^18.2^/BGC823 xenograft tumor and patients with gastric cancer and AEG	The radiotracer ^99m^Tc-PHG102 has shown high tumor uptake in both *ex vivo* and *in vivo* SPECT imaging studies. Preliminary clinical trials have also demonstrated its favorable safety profile and efficacy	Bai et al.^89^
NIRF	Cy5.5-5C9/IgG FD1080-5C9	mAb	gastric cancer	BGC823^CLDN18.2^/BGC823 xenograft tumor	Cy5.5-5C9 fluorescence imaging delineated subcutaneous CLDN18.2-positive tumors. FD1080-5C9 can be an NIR-II probe for surgical guidance	Zhao et al.^78^

AEG, adenocarcinoma of esophagogastric junction; ICG, indocyanine green; SUV, standard uptake value.

Following rigorous screening, 13 high-quality studies meeting predefined methodological criteria were identified. These studies were categorized by imaging modality and systemically analyzed for advancements in technology, diagnostic performance, and current limitations of CLDN18.2-targeted imaging. The review also highlights translational challenges to clinical adoption and outlines strategic recommendations to enhance the development and application of CLDN18.2-targeted molecular imaging, providing evidence-based direction for future research.

### immunoPET imaging

ImmunoPET represents a revolutionary advancement in molecular imaging, combining the high specificity of antibody-based targeting with the sensitivity and quantitative capabilities of PET.^16^ In efforts to assess CLDN18.2 expression, researchers have engineered three primary classes of immunoPET tracers: mAbs, single-domain antibodies (sdAbs), and peptides. This multimodal approach enables real-time, spatially resolved quantification of biomarker expression, facilitating both molecular diagnostics precision and therapeutic response assessment.

#### Monoclonal antibody

mAbs offer high affinity, stability, and specificity, which make them an attractive platform for targeted molecular imaging. However, their high molecular weight results in slow systemic clearance, needing extended waiting periods to achieve optimal tumor-to-background contrast.^90^ Consequently, mAb-based immunoPET probes are often paired with long-lived radioisotopes, such as ^89^Zr (half-life 3.3 days) and ^124^L (half-life 4.2 days), enabling imaging over prolonged time frames.^91^^,^^92^

**Figure 3:**
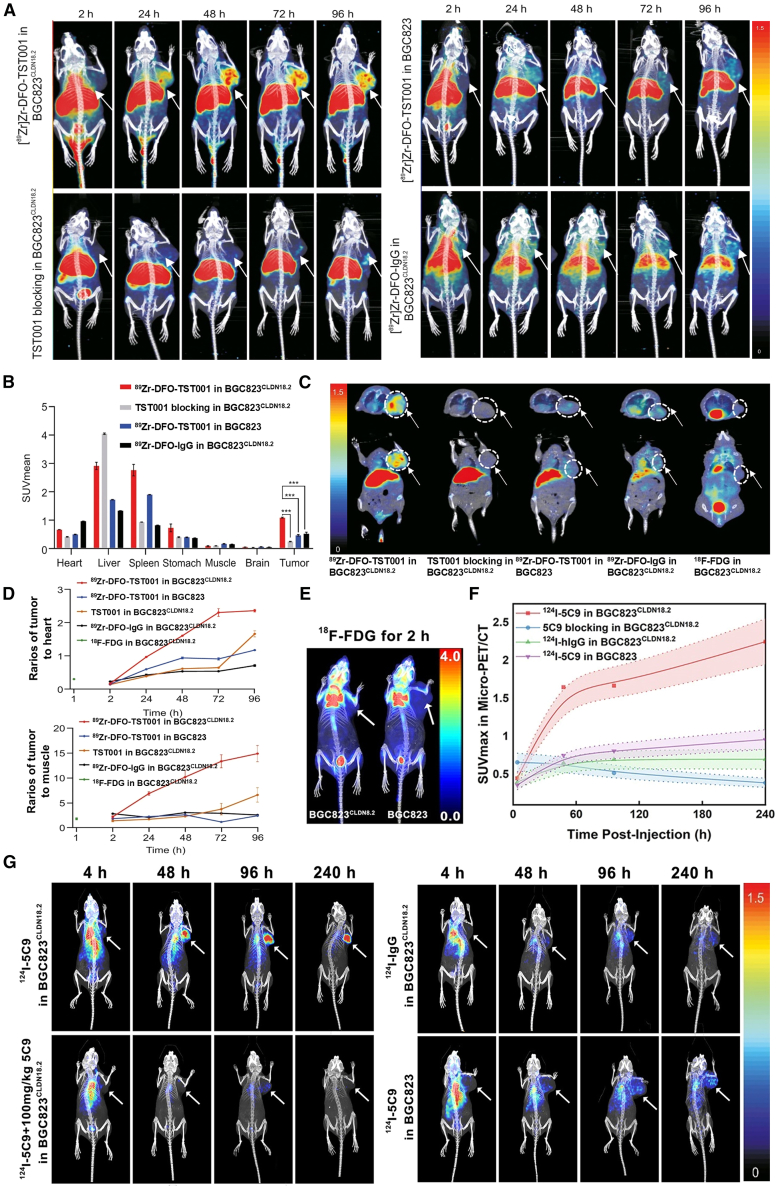
Monoclonal antibodies for CLDN18.2-targeted immunoPET imaging (A and B) (A) Representative PET images and (B) corresponding organs SUVmean values in BGC823^CLDN18.2^ and BGC823 xenograft models injected with ^89^Zr-DFO-TST001 or ^89^Zr-DFO-IgG. (C) Tumor uptake comparison between ^89^Zr-DFO-TST001 at 48 h post-injection (p.i.) and ^18^F-FDG at 1 h p.i. in BGC823^CLDN18.2^ mice. (D) Tumor-to-heart and tumor-to-muscle SUV ratios at various time points p.i. Reproduced under the terms of the CC-BY 4.0 license.^77^ Copyright 2023, Elsevier. (E) PET/CT images of ^18^F-FDG in BGC823^CLDN18.2^ (left) and BGC823 (right) tumor-bearing mice. (F and G) (F) Tumor SUVmax curves and (G) PET images of BGC823^CLDN18.2^ and BGC823 xenograft models injected with ^124^I-5C9 or ^124^I-IgG. Reproduced with permission.^78^ Copyright 2023, American Chemical Society.

Chen et al.^77^ developed ^89^Zr-DFO-TST001, a CLDN18.2-targeted immunoPET tracer generated by conjugating the humanized mAb TST001 with ^89^Zr via the DFO chelator. ^89^Zr is cyclotron-produced through proton or deuteron bombardment of ^89^Y, which requires relatively lower proton energies, making production more accessible.^93^ Due to its favorable positron emission properties, ^89^Zr enables high-resolution PET imaging. The ^89^Zr-DFO-TST001 probe was evaluated in murine models bearing either CLDN18.2-positive (BGC823^CLDN18.2^) or CLDN18.2-negative (BGC823) tumors (Figure 3A). PET imaging demonstrated significantly higher tracer uptake in CLDN18.2-positive tumors compared to negative controls (Figure 3B). Furthermore, uptake remained consistently elevated throughout the observation period and exceeded that of the non-targeted control tracer (^89^Zr-DFO-IgG), confirming its specificity and sensitivity. Furthermore, the novel probe showed superior tumor uptake (Figure 3C) and higher tumor-to-background ratios (Figure 3D) than ^18^F-FDG, indicating enhanced specificity for CLDN18.2-positive lesions. However, substantial non-specific uptake in the liver and spleen was observed, likely due to off-target binding and hepatobiliary clearance, which may compromise diagnostic accuracy. To mitigate these limitations, probe design can be optimized, such as using antibody fragments like Fab and F(ab’)2 to prepare probes, or genetically engineering the Fc region of IgG.^94^^,^^95^ Another strategy is to replace radioactive isotopes, such as ^124^I, which will quickly be lost from the liver and spleen.^29^

Compared to ^89^Zr, ^124^L provides advantages such as improved accessibility, simplified synthesis, and efficient labeling protocols.^92^ Its nonpolar metabolites are rapidly cleared via hepatobiliary pathways, reducing background signal from prolonged tissue retention. Zhao et al.^78^ radiolabeled the anti-CLDN18.2 mAb 5C9 with ^124^I to generate a promising immunoPET probe for detecting CLDN18.2-positive tumors (Figure 3G). Notably, ^18^F-FDG uptake showed minimal differentiation between CLDN18.2-positive and -negative tumors (Figure 3E), whereas ^124^I-5C9 exhibited rapid and preferential accumulation in CLDN18.2-positive lesions within 96 h, as reflected by significantly higher SUVmax values (Figure 3F). These results support the potential of ^124^I-5C9 as a clinically translatable agent for the non-invasive detection and spatial mapping of CLDN18.2-expressing tumors.

Wang et al.^79^ constructed the first-in-human CLDN18.2-targeted PET tracer, ^124^I-18B10(10L), by radiolabeling the full-length antibody 18B10(10L) with ^124^I. In this preliminary clinical study, the tracer demonstrated excellent safety and specificity in detecting CLDN18.2-overexpressing tumors. Untreated lesions showed significantly higher 24-h uptake compared to lesions previously exposed to CLDN18.2-targeted therapies. Tracer uptake varied markedly across metastatic sites, with ovarian metastases exhibiting the highest uptake and lung metastases the lowest. No correlation was found between ^124^I-18B10(10L) and ^18^F-FDG uptake, confirming its unique capability for non-invasive CLDN18.2 expression profiling. However, limited tumor penetration due to the antibody’s large molecular size may cause discrepancies between tracer uptake and actual target expression. Future studies should prioritize smaller molecular probes, such as antibody fragments or engineered scaffolds, to improve tumor penetrability, accelerate imaging workflows, and enhance clinical applicability.^96^ In addition, patients must receive prophylactic iodine to block thyroid uptake before and during ^124^I imaging, which imposes logistical challenges and may hinder patient compliance and widespread clinical adoption.^78^

#### sdAb

**Figure 4:**
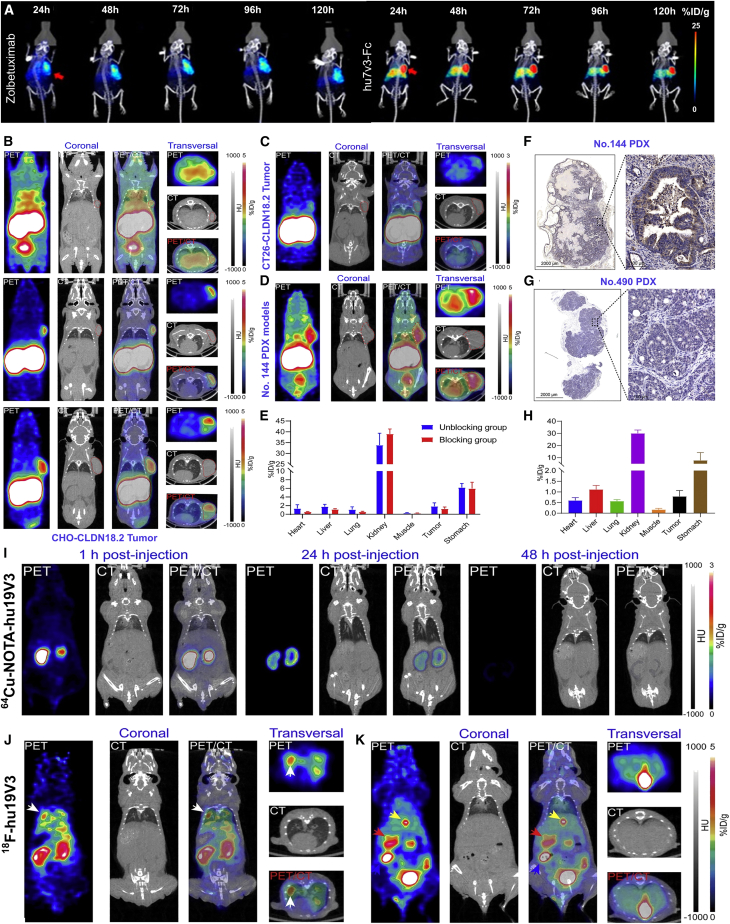
PET imaging performance of the hu7v3-Fc probe (A) Representative PET/CT images of SNU-620 tumor-bearing mice injected with either ^89^Zr-zolbetuximab or ^89^Zr-hu7v3-Fc. Reproduced under the terms of the CC-BY 4.0 license.^80^ Copyright 2022, Frontiers Media. (B–D) ^68^Ga-NOTA-hu19V3 successfully detected CLDN18.2-expressing tumors in (B) CHO-CLDN18.2, (C) CT26-CLDN18.2, and (D) no. 144 PDX models. (E–G) (E) ROI analysis of tracer uptake in the CHO-CLDN18.2 model. IHC analysis to confirm CLDN18.2 expression in no. 144 (F) versus No. 490 (G) PDX tumors. (H) ROI analysis of tracer uptake in the CT26-CLDN18.2 model. (I) Time-course PET imaging with ^64^Cu-NOTA-hu19V3. (J) ^18^F-hu19V3 immunoPET detected a disseminated CHO-CLDN18.2 lesion in the left lung. (K) ^18^F-hu19V3 exhibited significant gastric uptake and dual clearance via urinary and hepatobiliary pathways. ROI, region of interest. Reproduced with permission.^82^ Copyright 2022, Elsevier.

sdAbs, also known as nanobodies, represent a distinct class of antibody fragments composed solely of the variable domain of heavy-chain-only antibodies (VHHs).^97^ With a molecular weight of just 12–15 kDa, sdAbs are substantially smaller than conventional antibodies and their fragments. This compact size confers several advantages, such as excellent water solubility, remarkable thermal and chemical stability, strong antigen-binding affinity, intrinsic anti-tumor activity, and superior tissue penetration.^98^ Zhong et al.^80^ demonstrated the superior targeting performance of a humanized bispecific sdAb, hu7v3-Fc, compared to zolbetuximab in CLDN18.2-positive tumors. Flow cytometry and IHC analyses revealed that hu7v3-Fc selectively bound to differentiated epithelial cells of the gastric mucosa, thereby emphasizing the high specificity of hu7v3-Fc for CLDN18.2. ImmunoPET imaging conducted in an SU-620 xenograft model revealed that ^89^Zr-hu7v3-Fc achieved faster tumor uptake and superior tumor penetration than ^89^Zr-zolbetuximab (Figure 4A). Quantitative analysis further showed improved tumor-to-liver and tumor-to-muscle ratios for hu7v3-Fc, underscoring the pharmacokinetic and structural advantages of sdAbs over full-length mAbs. Consequently, sdAbs have emerged as a promising molecular scaffold for the development of CLDN18.2-targeted immunoPET probes.

Hu et al.^81^ engineered three ^89^Zr-labeled CLDN18.2-targeted sdAb tracers for molecular imaging in gastric cancer models, including ^89^Zr-VHH, ^89^Zr-VHH-ABD (albumin-binding domain), and ^89^Zr-VHH-Fc. Among these, ^89^Zr-VHH-Fc had the highest tumor uptake but also exhibited elevated liver background. In contrast, ^89^Zr-VHH-ABD achieved a favorable balance between tumor accumulation and liver signal, with enhanced tissue penetration and reduced non-specific uptake, making it the most promising candidate. This study highlights the potential of optimizing antibody formats by integrating the benefits of nanobodies and Fc-fusion constructs. However, the use of PC3 cells as negative controls, despite their low but detectable CLDN18.2 expression, may have introduced variability, underscoring the need for stricter negative controls or orthogonal validation strategies in future studies.

In addition to ^89^Zr, Wei et al.^82^ explored multiple radionuclides (^68^Ga, ^64^Cu, and ^18^F) to develop and evaluate CLDN18.2-targeted molecular imaging tracers in mouse models. Due to its short half-life (67.6 min), ^68^Ga is well suited for imaging with rapidly clearing, low-molecular-weight probes, such as sdAbs.^99^ Its generator-based production also facilitates widespread clinical application. Using the ^68^Ga-NOTA-hu19V3 tracer, the team conducted PET imaging in three subcutaneous tumor models: CHO-CLDN18.2 (Figure 4B), CT26-CLDN18.2 (Figure 4C), and CLDN18.2-positive PDX no. 144 (Figure 4D). The probe rapidly identified CLDN18.2-positive tumors, with notably higher uptake in the No. 144 PDX model, likely due to its naturally high CLDN18.2 expression (Figures 4F and 4G). However, substantial renal accumulation was observed (Figures 4E–4H), a common limitation for sdAbs due to their small size and rapid renal clearance. To address this issue, extending sdAb circulation time via Fc or albumin-binding domain fusion has been proposed.^94^^,^^95^ To further address renal background and enable delayed imaging, the team employed radioisotope ^64^Cu (half-life 12.7 h) to label the NOTA-hu19V3 probe, which showed reduced kidney uptake and improved contrast in later imaging time points (Figure 4I).^100^ While ^64^Cu-NOTA-hu19V3 successfully delineated gastric tissue, it failed to detect disseminated CHO-CLDN18.2 lesions, potentially due to insufficient early sensitivity or poor tumor engraftment.

Given the limited availability and high cost of ^64^Cu, researchers also utilized ^18^F-labeled hu19V3 (half-life 109.8 min).^101^ The ^18^F-hu19V3 probe effectively detected CHO-CLDN18.2 metastases in the lungs (Figure 4J) and exhibited dual clearance via both the urinary and hepatobiliary pathways (Figure 4K), in contrast to the predominantly renal excretion seen with ^68^Ga and ^64^Cu tracers. Collectively, these studies demonstrate that CLDN18.2-targeted PET tracers can effectively visualize gastric and colorectal tumors, with ^68^Ga- and ^18^F-labeled probes demonstrating complementary advantages based on isotope-specific pharmacokinetics and imaging workflows.

**Figure 5:**
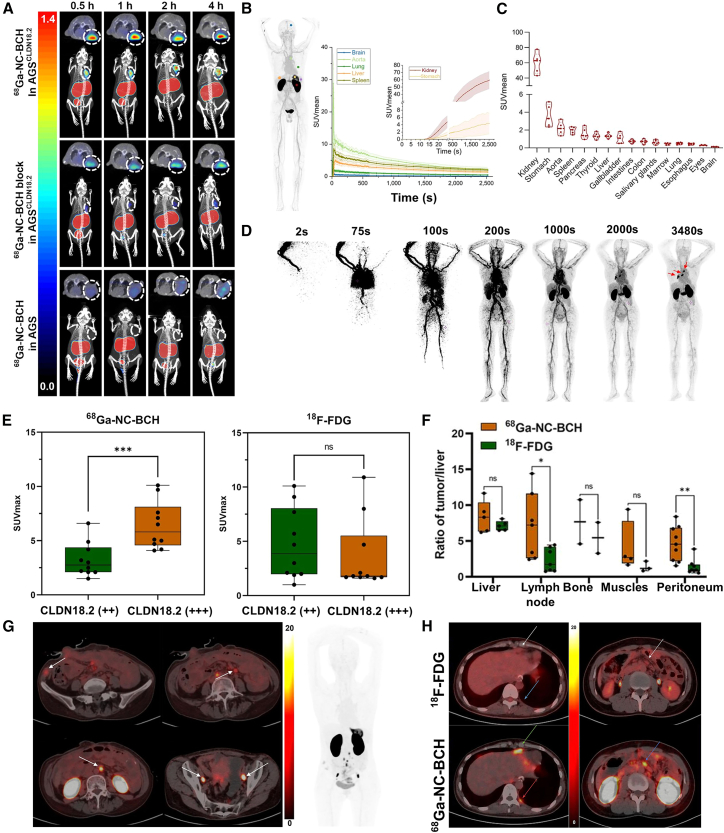
^68^Ga-NC-BCH PET/CT imaging in preclinical models and patients (A) ^68^Ga-NC-BCH PET images of AGS^CLDN18.2^ and CLDN18.2-negative AGS xenograft models. (B) Time-activity curves depicting SUVmean dynamics in selected organs of the patient. (C) ^68^Ga-NC-BCH uptake in different organs at 42 min post-injection. (D) Dynamic PET/CT with ^68^Ga-NC-BCH to map temporal radiotracer distribution in tumors. (E) Boxplot comparing SUVmax values of ^68^Ga-NC-BCH and ^18^F-FDG in lesions from 11 patients stratified by CLDN18.2 expression (++ and +++). (F) Comparison of tumor-to-non-tumor ratio between ^68^Ga-NC-BCH and ^18^F-FDG in metastatic lesions. (G) Multiple lymph node metastases in a patient imaged with ^68^Ga-NC-BCH PET. (H) Detection of pleural (left) and abdominal (right) metastases by ^68^Ga-NC-BCH PET/CT that were inconspicuous on ^18^F-FDG PET/CT. Reproduced under the terms of the CC-BY 4.0 license.^83^ Copyright 2024, Society of Nuclear Medicine and Molecular Imaging.

Clinical investigations employing sdAb-based immunoPET imaging have been initiated in patients with gastrointestinal cancer. This translational approach utilizes engineered molecular probes to achieve antigen-specific visualization, representing a critical step from bench to bedside in oncologic molecular imaging. For example, Qi et al.^83^ developed ^68^Ga-NC-BCH, a novel PET probe designed for rapid assessment of CLDN18.2 expression in patients with gastrointestinal cancer. In preclinical models, tracer uptake in CLDN18.2-positive AGS xenograft tumors was significantly higher than in CLDN18.2-negative controls (Figure 5A). Subsequently, whole-body dynamic PET/CT scans in patients revealed rapid systemic distribution of the probe, followed by clearance from most organs except the stomach and kidneys (Figure 5B). At 1-h post-injection, tracer uptake in most normal tissues was minimal, whereas significant retention was observed in the stomach and kidneys (Figure 5C). Importantly, ^68^Ga-NC-BCH demonstrated excellent specificity, with SUVmean values in CLDN18.2-positive lesions increasing over time (Figure 5D) and SUVmax values correlating strongly with CLDN18.2 expression levels (Figure 5E). Intrapatient heterogeneity in CLDN18.2 expression was also observed among metastatic sites (Figure 5G). Compared to the standard ^18^F-FDG PET/CT, ^68^Ga-NC-BCH exhibited significantly higher tumor-to-nontumor (T/NT) ratios, particularly in lymph node and peritoneal metastases (Figure 5F), and was capable of detecting small peritoneal and pleural lesions undetected by ^18^F-FDG (Figure 5H). These findings position ^68^Ga-NC-BCH PET/CT as a promising molecular imaging tool for patient stratification and longitudinal response monitoring in CLDN18.2-targeted precision medicine.

Wang et al.^84^ developed ^68^Ga-PMD22, a novel nanobody-based PET tracer targeting CLDN18.2 for the detection of gastrointestinal tumors, and conducted both preclinical and clinical evaluations of this agent. The probe exhibited 6.7-fold greater binding affinity to CLDN18.2-positive cells compared to negative controls. In xenograft models, ^68^Ga-PMD22 PET/CT imaging showed rapid tumor accumulation, with peak uptake observed at 1-h post-injection, resulting in higher SUV and tumor-to-background ratios than ^18^F-FDG. In a clinical study involving 17 patients, the tracer achieved a diagnostic accuracy of 93.3%, with strong concordance between SUVmax and CLDN18.2 expression assessed by IHC. These findings support ^68^Ga-PMD22 as an effective agent for theranostic applications in CLDN18.2-positive gastrointestinal tumors.

However, both ^68^Ga-PMD22 and ^68^Ga-NC-BCH exhibited physiological uptake in normal human gastric mucosa, which may affect diagnostic specificity. To mitigate nonspecific renal and gastric retention, several potential strategies have been considered, including the incorporation of cleavable linkers between nanobodies and radionuclides, co-administration of renal uptake inhibitors, and competitive blockade of CLDN18.2 receptors in the gastric mucosa.

#### Peptides

**Figure 6:**
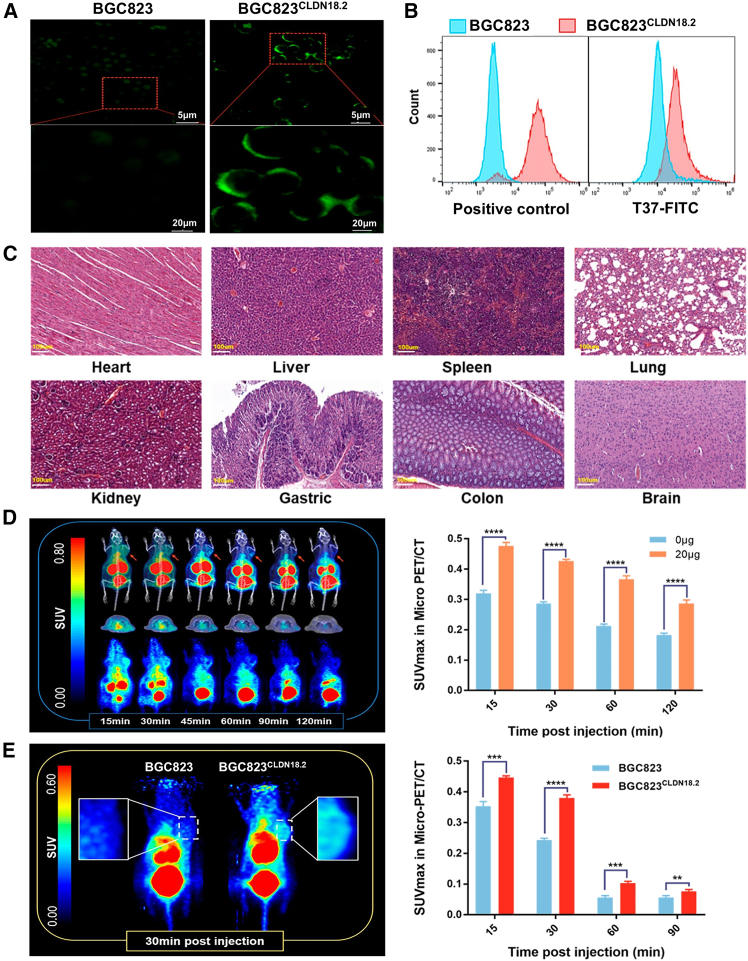
^68^Ga-DOTA-T37 selectively targets CLDN18.2-expressing tumors in murine models (A) Immunofluorescence imaging using FITC-labeled T37 peptide in BGC823 and BGC823^CLDN18.2^ cells. (B) Flow cytometry analysis confirming T37 binding specificity. (C) H&E staining of major organs demonstrating biosafety. (D) PET/CT images and ^68^Ga-DOTA-T37 uptake analysis in CLDN18.2-expressing protein model. (E) *In vivo* PET/CT imaging and biodistribution of ^68^Ga-DOTA-T37 in BGC823 and BGC823^CLDN18.2^ tumor-bearing mice. Reproduced with permission.^85^ Copyright 2023, American Chemical Society.

Peptide-based probes offer excellent selectivity, biocompatibility, tissue penetration, and favorable pharmacokinetics.^102^ These attributes position them as attractive alternatives to traditional antibody-based agents for cancer diagnostics, with growing evidence suggesting their potential to surpass antibodies in certain theranostic applications. Wang et al.^85^ utilized a phage display peptide screening approach to identify T37, a CLDN18.2-specific peptide, and subsequently developed ^68^Ga-DOTA-T37 as a targeted PET probe. *In vitro* validation confirmed the specificity and affinity of T37 for CLDN18.2 (Figures 6A and 6B). Hematoxylin-eosin (H&E) staining of major organs (Figure 6C) and human dosimetry estimates indicated a favorable safety profile. PET/CT imaging in CLDN18.2-expressing protein models (Figure 6D), as well as in BGC823 and BGC823^CLDN18.2^ xenograft-bearing mice (Figure 6E), demonstrated specific *in vivo* uptake in CLDN18.2-positive tissues. These findings support ^68^Ga-DOTA-T37 as a promising CLDN18.2-targeted radioligand with strong translational potential for CLDN18.2-targeted cancer theranostics. However, as a linear peptide, T37 is susceptible to enzymatic degradation, *in vivo* instability, and rapid metabolic clearance.^103^ Future efforts to improve its pharmacological performance may include chemical modifications such as peptide cyclization, D-amino acid incorporation, and C-terminal amidation to enhance proteolytic resistance and prolong systemic circulation.^104^^,^^105^

### ImmunoSPECT imaging

SPECT is a nuclear medicine imaging modality that utilizes gamma-ray-emitting radionuclides, such as technetium-99m (^99m^Tc) and iodine-123 (^123^I), to produce tomographic images.^106^ By quantifying the spatial distribution and intensity of gamma emissions, SPECT enables the reconstruction of detailed functional images. It provides unique insights into tissue perfusion and metabolic activity and has gained broad clinical adoption, particularly in oncologic diagnostics, due to its lower cost compared to PET imaging.^107^ Advancements in immunoSPECT have further enhanced the clinical utility of SPECT by incorporating the molecular specificity of antibodies. This approach enables simultaneous anatomical localization and molecular characterization of disease targets.^16^^,^^108^ However, the use of full-length antibodies introduces limitations such as reduced tissue penetration, prolonged circulation time, and elevated background signal, often requiring delayed imaging to optimize contrast.^108^

Zeng et al.^86^ developed ^177^Lu-TST001, a theranostic agent composed of a CLDN18.2-targeted mAb conjugated with the therapeutic radionuclide lutetium-177. With a half-life of 6.7 days, ^177^Lu emits both beta particles for cytotoxicity and gamma rays for imaging, making it well-suited for radioimmunotherapy (RIT) and companion diagnostics.^109^ In preclinical studies, ^177^Lu-TST001 demonstrated specific targeting toward CLDN18.2-positive gastric cancer cell lines and xenografts, with minimal uptake in CLDN18.2-negative controls. Using SPECT imaging, the probe enabled real-time visualization of tumor targeting, and subsequent therapeutic studies showed effective tumor suppression and a favorable safety profile. These results underscore the potential of ^177^Lu-TST001 as a dual-modality agent for both imaging and therapy of CLDN18.2-expressing gastric tumors. To mitigate hepatic and splenic off-target uptake, strategic antibody engineering could be prioritized, including the adoption of scFv or BsAb platforms to eliminate Fc-mediated nonspecific interactions while maintaining antigen-binding capacity. Complementary approaches involve implementing SPECT/CT-based biodynamic monitoring to guide adaptive dosing protocols through pharmacokinetic modeling.

**Figure 7:**
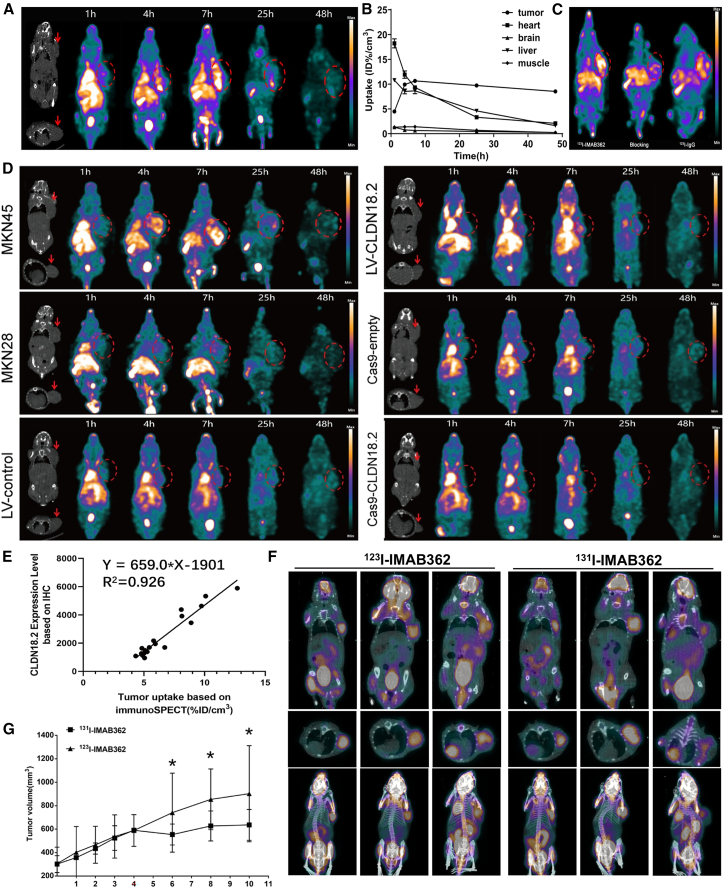
^131^I/^123^I-IMAB362 enables CLDN18.2-targeted SPECT imaging and radioimmunotherapy (A) ImmunoSPECT or SPECT/CT images after injection of ^123^I-IMAB362 in mice bearing MKN45 xenografts. (B) Biodistribution of ^123^I-IMAB362 in major organs. (C) ImmunoSPECT imaging of MKN45 xenografts with ^123^I-IMAB362, with and without excess unlabeled IMAB362, and control imaging using ^123^I-IgG. (D) ImmunoSPECT and SPECT/CT images of ^123^I-IMAB362 in mice bearing xenografts with varying CLDN18.2 expression levels. (E) Correlation between ^123^I-IMAB362 uptake (semi-quantified from 7-h SPECT/CT imaging) and IHC-derived CLDN18.2 expression levels. (F) Longitudinal ^18^F-FDG PET/CT imaging of patient-derived gastric tumor xenografts on days 1, 2, and 7 following intravenous administration of ^131^I-IMAB362 or ^123^I-IMAB362. (G) Tumor volumes in mice treated with ^131^I-IMAB362 or ^123^I-IMAB362. Reproduced with permission.^87^ Copyright 2024, American Chemical Society.

Wu et al.^87^ developed ^123^I/^131^I-IMAB362 as a theranostic pair for CLDN18.2-targeted SPECT/CT and RIT in gastric cancer. While ^124^I (half-life 4.2 days) serves as an excellent PET isotope, its clinical utility is limited by poor accessibility and high cost.^92^ In contrast, ^123^I and ^131^I are widely utilized in clinical theranostics, including the established use of ^123^I/^131^I-*meta*-iodobenzylguanidine (MIBG) for neuroblastoma management.^110^ ^123^I (half-life 13.2 h) emits gamma rays ideal for diagnostic imaging, offering high image resolution and reduced radiation burden,^111^ whereas ^131^I (half-life 8.06 days) emits beta particles for effective therapeutic applications, such as thyroid disease management and RIT.^112^ In their study, high accumulation of ^123^I-IMAB362 was observed in CLDN18.2-positive MKN45 xenografts, as evidenced by immunoSPECT imaging. Notable off-target uptake was also detected in the heart, liver, and gallbladder (Figures 7A and 7B). The probe demonstrated markedly higher uptake in MKN45 tumors compared to both the ^123^I-IgG control group and the CLDN18.2-blocking group, thereby confirming its target specificity (Figure 7C). Further imaging studies in diverse xenograft models, including CLDN18.2-negative MKN28, CLDN18.2-overexpressing, and CLDN18.2-knockout tumors (Figure 7D), revealed a strong correlation between ^123^I-IMAB362 tumor uptake and CLDN18.2 expression, consistent with IHC results (Figure 7E). Collectively, these data suggest that ^123^I-IMAB362 may serve as a predictive imaging biomarker for selecting suitable candidates for CLDN18.2-targeted therapies.

The therapeutic counterpart, ^131^I-IMAB362, significantly decreased SUVmax on ^18^F-FDG PET/CT in the CLDN18.2-positive PDX model of gastric cancer (Figure 7F), and produced a more pronounced antitumor effect than ^123^I-IMAB362 (Figure 7G). Although weight loss was noted in tumor-bearing mice, a gradual increase in weight was observed in healthy mice treated with ^123^I/^131^I-IMAB362, supporting its tolerability. These findings suggest that ^123^I/^131^I-IMAB362 may serve as a valuable isotopic pair targeting CLDN18.2 for imaging and therapy of gastric cancer. Nonetheless, certain limitations remain. Rapid tumor growth resulting in partial necrosis in xenografts may compromise imaging accuracy. Additionally, ^131^I-IMAB362 showed reduced *in vivo* stability compared to its ^123^I-labeled counterpart. Future studies should focus on improving stability and efficacy through alternative radionuclides, more robust chelating agents, or optimized antibody formats (e.g., Fab, BsAbs, scFv-Fc, and sdAbs).

In addition to ^123^I and ^131^I, Wang et al.^88^ utilized ^125^I-labeled Zolbertuximab to evaluate its specific binding affinity for CLDN18.2 and its potential in gastric cancer theranostics. ^125^I (half-life 60.1 days), with continuous low-energy gamma emission, is extensively utilized for radiolabeling macromolecular therapeutics.^113^ Its radiophysical properties also render it suitable for targeted radionuclide therapy applications. *In vitro* studies confirmed that ^125^I-Zolbertuximab exhibits high binding affinity toward MKN45-CLDN18.2 cells. The radiotracer demonstrates favorable tumor retention kinetics coupled with rapid systemic clearance as evidenced by SPECT/CT imaging. However, progressive thyroidal uptake observed during longitudinal imaging indicated an *in vivo* deiodination process and metabolic instability. To improve radiotracer stability, further development of indirect labeling strategies utilizing auxiliary groups is warranted.

**Figure 8:**
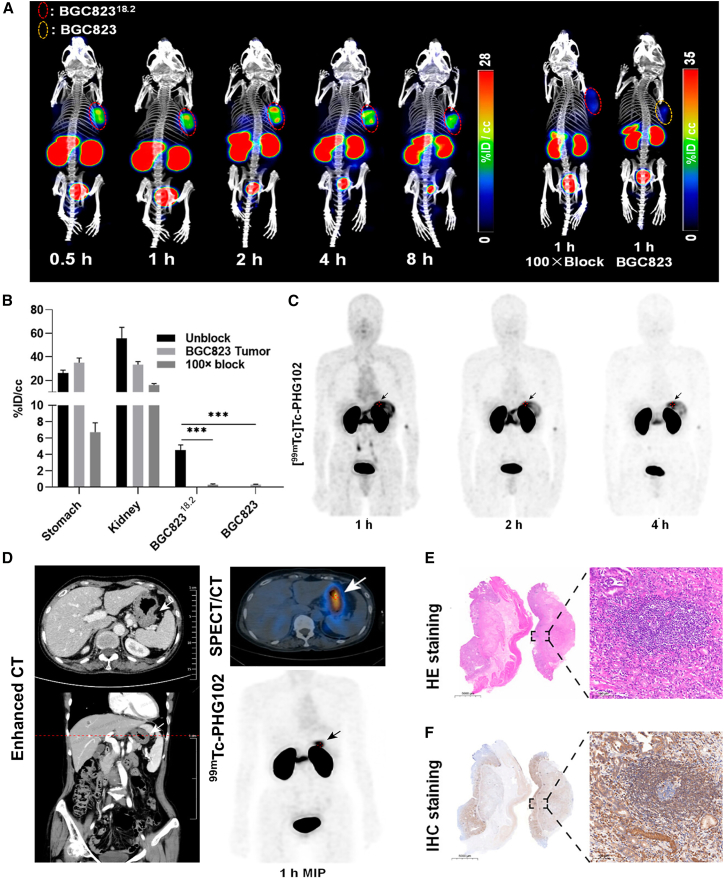
^99m^Tc-PHG102 immunoSPECT in CLDN18.2-positive cancer (A) SPECT/CT imaging of ^99m^Tc-PHG102 in BGC823^CLDN18.2^ and BGC823 tumor-bearing mice. (B) Tumor and organ uptake across groups. (C) MIP images of ^99m^Tc-PHG102 SPECT/CT of a 62-year-old male AEG patient. (D) ^99m^Tc-PHG102-enhanced CT, SPECT, and fused SPECT/CT images in a gastric cancer patient. (E and F) (E) H&E and (F) IHC staining of CLDN18.2 in the tumor lesion. Reproduced with permission.^89^ Copyright 2024, American Chemical Society. AEG, adenocarcinoma of esophagogastric junction; MIP, maximal intensity projection.

Due to the prolonged circulation and metabolism of full-length mAbs, their efficacy may be limited in scenarios requiring rapid tumor targeting or clearance.^90^ To address this, Bai et al.^89^ developed a novel radiolabeled nanobody, ^99m^Tc-PHG102, for CLDN18.2-targeted imaging in gastric cancer. Technetium-99m (^99m^Tc), generated from a ^99^Mo-^99m^Tc generator, is widely used in SPECT imaging due to its ideal characteristics: high target specificity, low systemic toxicity, monoenergetic gamma emission, and a short half-life (6.02 h) that aligns well with nanobody pharmacokinetics.^114^ SPECT/CT imaging showed that ^99m^Tc-PHG102 rapidly and specifically accumulated in BGC823^CLDN18.2^ xenograft tumors (Figure 8A) and was efficiently cleared via renal excretion (Figure 8B). In addition, SPECT with ^99m^Tc-PHG102 can detect CLDN18.2-positive gastric and esophagogastric junction cancer in patients (Figures 8C and 8D), consistent with H&E and IHC staining (Figures 8E and 8F). These findings highlight the potential of ^99m^Tc-PHG102 immunoSPECT as an effective tool for assessing CLDN18.2 expression and identifying eligible patients for targeted therapies.

Nonetheless, physiological CLDN18.2 expression in normal gastric mucosa may lead to elevated background signals, potentially limiting diagnostic accuracy. This limitation could be mitigated by PET/CT, which offers enhanced spatial resolution and detection sensitivity. Furthermore, high renal clearance of nanobody probes may result in substantial kidney uptake, potentially affecting the image quality of adjacent tissues. Future work should focus on optimizing probe design or co-administering agents that reduce renal uptake to improve imaging quality.

### NIRF imaging

NIRF imaging is an optical imaging technique that utilizes NIR light (wavelength 700–1700 nm), spanning the NIR-I and NIR-II spectral windows.^115^ This modality uses specific fluorescent probes, such as indocyanine green and cyanine, that emit fluorescence under near-infrared light excitation. The emitted signals are captured through high-sensitivity detectors to generate images. NIRF imaging offers several advantages, including minimal background autofluorescence, lack of ionizing radiation, and the capacity for real-time, dynamic detection.^116^ As a result, it has emerged as a valuable tool in tumor diagnosis and intraoperative surgical guidance.

**Figure 9:**
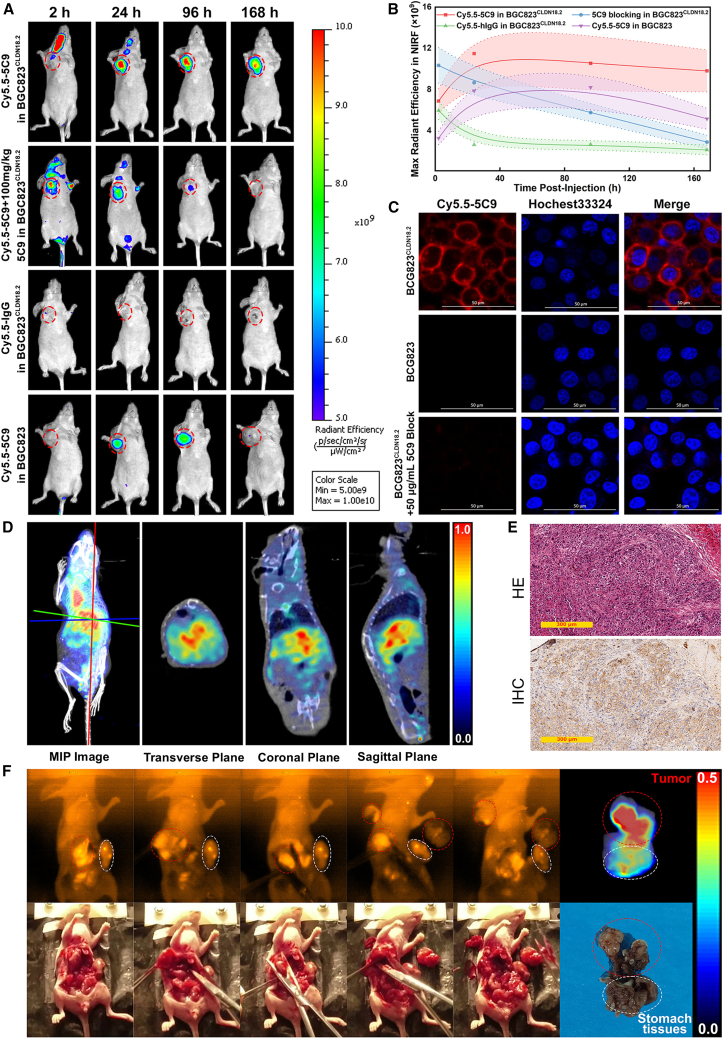
NIRF probes in CLDN18.2-targeted imaging (A) NIRF imaging comparison of mice bearing BGC823^CLDN18.2^ and BGC823 tumors following injection with Cy5.5-5C9 or control IgG. (B) Quantification of Cy5.5 fluorescence signal across four tumor groups. (C) IF staining of BGC823^CLDN18.2^ and BGC823 cells using Cy5.5-5C9 and nuclear counterstain Hoechst 33342. (D) PET/CT imaging of orthotopic BGC823^CLDN18.2^ gastric tumors using ^124^I-5C9. (E) H&E/IHC analysis of orthotopic BGC823^CLDN18.2^ gastric tumor tissues. (F) NIR-II fluorescence imaging with FD1080-5C9 tracer. Reproduced with permission.^78^ Copyright 2023, American Chemical Society. IF, immunofluorescence.

Zhao et al.^78^ developed an NIRF probe, Cy5.5-5C9, by conjugating the CLDN18.2-targeted mAb 5C9 with the fluorescent dye Cyanine5.5 (Cy5.5). They performed NIRF imaging on mice bearing CLDN18.2-positive or CLDN18.2-negative tumors (Figure 9A). Quantitative imaging analysis demonstrated significantly elevated fluorescence signal intensity in CLDN18.2-overexpressing lesions compared to non-target tissues (Figure 9B). Flow cytometry further validated the target specificity of Cy5.5-5C9 in isogenic cell lines (Figure 9C). To enhance intraoperative imaging, they used FD1080, an NIR-II fluorescent dye, to construct the FD1080-5C9 probe. Mice bearing CLDN18.2-positive xenografts were first imaged using ^124^I-5C9 immunoPET (Figure 9D), followed by intraoperative NIR-II fluorescence imaging with FD1080-5C9 (Figure 9F). H&E and IHC analyses confirmed high CLDN18.2 expression in excised tumors, supporting tracer specificity and model relevance (Figure 9E). These results underscore the potential of NIRF imaging probes for precise localization and fluorescence-guided surgery in CLDN18.2-positive malignancies.

Further translational and clinical studies are warranted to validate their utility in human applications. However, the clinical translation of NIRF imaging probes from preclinical models to human applications faces critical technical barriers. The primary challenge is that the substantial depth of human tissues compared to murine models necessitates enhanced penetration capacity of NIR probes. Some endogenous interference sources in humans can also reduce the tumor-to-background signal ratio. To address these technical limitations, future research can focus on developing ultra-narrowband filters to improve the signal-to-noise ratio and optimize probe-specific uptake at targets.^117^

### Conclusion and future outlook

CLDN18.2 has emerged as a promising therapeutic target for digestive system malignancies, garnering significant attention in precision oncology. Although zolbetuximab has received FDA approval for treating gastric cancer, its clinical performance is limited by suboptimal efficacy in patients with low CLDN18.2 expression. To overcome these limitations, next-generation strategies, such as BsAbs, which harness multi-pathway synergy, and ADCs, which enable targeted cytotoxic payload delivery, are under active investigation. However, several challenges hinder the clinical translation of CLDN18.2-targeted therapies. First, safety concerns, including CRS with CAR-T cell therapies and off-target toxicity of ADC payloads, necessitate a comprehensive toxicology assessment.^67^ Second, BsAb development must address technical complexities related to antibody engineering, stability, and manufacturing scalability.^118^ Additionally, current diagnostic approaches, primarily reliant on IHC, suffer from procedural invasiveness and interpretive variability, limiting dynamic and quantitative assessment of CLDN18.2 expression.^14^

Molecular imaging offers a non-invasive and precise method for profiling CLDN18.2 expression, complementing tissue-based diagnostics. Additionally, it enables patient stratification, real-time intraoperative guidance, prognostic evaluation, and monitoring of therapeutic response. Among imaging modalities, NIRF imaging provides real-time visualization without radiation exposure, but is limited by lower sensitivity and depth penetration. Nuclear medicine imaging techniques, including PET and SPECT, offer higher sensitivity and unlimited tissue penetration depth. In particular, PET stands out for its superior imaging performance, despite higher costs compared to SPECT. In terms of standardization and reproducibility of imaging modalities, the metabolic dynamics of PET tracers are affected by the patient’s metabolic status.^119^ SPECT attenuation correction is prone to errors in heterogeneous tissues, and the modulation of excitation light intensity during NIRF navigation can cause excessive fluctuations in fluorescence intensity measurements of the same lesion. Notably, multimodal imaging strategies synergistically integrate complementary strengths from diverse modalities, offering transformative potential in addressing the challenges of invasiveness, spatial resolution limitations, and economic constraints.

The advancement of CLDN18.2-targeted imaging modalities faces challenges due to inherent limitations in tracer characteristics, including stability, target specificity, tissue penetration capabilities, and pharmacokinetic profiles. In terms of probe design, full-length mAbs provide high specificity but are hindered by slow clearance and delayed imaging windows. In contrast, smaller molecules, including BsAbs, nanobodies, and peptides, offer faster kinetics and better tissue penetration, making them attractive candidates for future development. Despite encouraging results in preclinical and early clinical studies, several obstacles remain. Most CLDN18.2-targeted imaging studies have used transfected cell lines or PDX models due to the scarcity of naturally and stably expressing CLDN18.2 cancer cell lines. Moreover, control groups are often suboptimal, consisting of low-expression rather than truly negative models. In addition, probe stability, such as the deiodination observed with ^125^I-labeled zolbertuximab, poses limitations on *in vivo* performance. Future work should focus on enhancing probe stability using alternative radiometals, auxiliary groups, optimized chelators, and antibody fragments.

A key challenge in nanobody-based imaging is off-target uptake in normal gastric mucosa and kidneys, which compromises image contrast. Several methods may mitigate this, including Fc fusion with albumin-binding domains or IgG to modulate pharmacokinetics, use of long-lived radionuclides for delayed imaging, cleavable linkers to facilitate clearance, and metabolic inhibitors (e.g., protamine, sodium malate, and fructose) to reduce renal uptake. Additionally, CLDN18.2 receptor blocking with unlabeled nanobodies may reduce non-specific binding in the stomach. Optimizing the pharmacokinetics and biodistribution of imaging agents remains essential to improving target-to-background ratios. Future studies should systematically evaluate these approaches through rigorous preclinical validation and clinical translation, with an emphasis on safety, specificity, and potential synergy with existing therapies. These efforts will accelerate the development of effective theranostic strategies for CLDN18.2-positive malignancies, ultimately enhancing diagnostic accuracy and therapeutic outcomes.

### Data and code availability

Data will be made available on request.

## Acknowledgments

This work was supported by the 10.13039/100007015University of Wisconsin-Madison and the 10.13039/100000002National Institutes of Health (P30 CA014520), the 10.13039/501100001809National Natural Science Foundation of China (82472018 and 82171970), 10.13039/501100005090Beijing Nova Program (20240484725), 10.13039/501100009592Beijing Municipal Science & Technology Commission (Z221100007422027), 10.13039/501100012166National Key Research and Development Program of China (2024YFE0113500), and the National High Level Hospital Clinical Research Funding (Interdisciplinary Research Project of Peking University First Hospital, 2023IR17 and 2024IR07; Scientific and Technological Achievements Transformation Incubation Guidance Fund Project of Peking University First Hospital, 2024CX18).

## Author contributions

Y.L.: conceptualization, writing – original draft, and writing – review & editing; W.H.: writing – original draft and writing – review & editing. J.C.H.: writing – review & editing. Z.S.: supervision and writing – review & editing. W.C.: conceptualization, supervision, writing – review & editing, and funding acquisition. L.K.: conceptualization, supervision, writing – review & editing, and funding acquisition.

## Declaration of interests

W.C. declares conflict of interest with the following corporations: Portrai, Inc.; rTR Technovation Corporation; Four Health Global Pharmaceuticals Inc.; and POP Biotechnologies, Inc.
